# Motor (but not auditory) attention affects syntactic choice

**DOI:** 10.1371/journal.pone.0195547

**Published:** 2018-04-16

**Authors:** Mikhail Pokhoday, Christoph Scheepers, Yury Shtyrov, Andriy Myachykov

**Affiliations:** 1 Centre for Cognition and Decision Making, National Research University Higher School of Economics, Russian Federation; 2 Institute of Neuroscience and Psychology, University of Glasgow, Glasgow, United Kingdom; 3 Centre of Functionally Integrative Neuroscience (CFIN), Department of Clinical Medicine, Aarhus University, Denmark; 4 Department of Psychology, Northumbria University, Newcastle-upon-Tyne, United Kingdom; Open University, UNITED KINGDOM

## Abstract

Understanding the determinants of syntactic choice in sentence production is a salient topic in psycholinguistics. Existing evidence suggests that syntactic choice results from an interplay between linguistic and non-linguistic factors, and a speaker’s attention to the elements of a described event represents one such factor. Whereas multimodal accounts of attention suggest a role for different modalities in this process, existing studies examining attention effects in syntactic choice are primarily based on visual cueing paradigms. Hence, it remains unclear whether attentional effects on syntactic choice are limited to the visual modality or are indeed more general. This issue is addressed by the current study. Native English participants viewed and described line drawings of simple transitive events while their attention was directed to the location of the agent or the patient of the depicted event by means of either an *auditory* (monaural beep) or a *motor* (unilateral key press) lateral cue. Our results show an effect of cue location, with participants producing more passive-voice descriptions in the patient-cued conditions. Crucially, this cue location effect emerged in the motor-cue but not (or substantially less so) in the auditory-cue condition, as confirmed by a reliable interaction between *cue location* (agent vs. patient) and *cue type* (auditory vs. motor). Our data suggest that attentional effects on the speaker’s syntactic choices are modality-specific and limited to the visual and motor, but not the auditory, domain.

## Introduction

### Multimodal attention and syntactic choice

Describing visually perceived events requires a number of decisions by the speaker, including the selection of words, assignment of thematic roles, and specification of syntactic structure. One aspect of those decisions is structural, such as the specific syntactic choice between active and passive voice in English sentence production. Multiple studies (see [1] for a recent review) showed that directing the speaker’s attention to one of the event’s referents by means of *visual* cueing biases subject role assignment to the cued referent, leading to the subsequent choice between the available structural alternatives. For example, speakers tend to select the active-voice frame when their attention is cued towards the event’s agent and they tend to select the passive-voice frame when their attention is cued to the patient. It remains unclear, however, whether this correspondence between attentional focus and syntactic choice is limited to the visual modality and, correspondingly, to the use of visual cues. At least hypothetically, the underlying link between attention and grammar may be more general and, therefore, modality-independent. The latter is intuitively plausible for two reasons. First, in real-life conversations, we tend to not only talk about visually perceived aspects of described events. In fact, relevant sensory information about an event can originate from any sensory modality or even from multiple modalities simultaneously. The resulting syntactic choice may therefore reflect an integrated attentional bias resulting from signals from different sensory inputs. Second, studies on multimodal integration show convincingly that inputs originating from different sensory modalities converge in an interactive manner (e.g. [2–4]). Therefore, it is possible that attentional cues originating from other modalities can lead to a similar mapping between the cued referent and its constituent role in the sentence’s syntactic structure. Here, we present results of a study, in which native speakers of English engaged in a picture-description sentence production task while their attention was directed towards one of the two protagonists of a transitive event by means of *motor* and *auditory* lateral cues. Before introducing the study in more detail, we will briefly review the available evidence.

#### Visual attention and sentence production

Several existing studies used variants of the visual cueing paradigm [5] in order to demonstrate that syntactic choice, at least in part, reflect the distribution of the speaker’s visual attention across the elements of the described scene [6–16]. A recurrent finding in these studies is that the visually cued element tends to be selected as the syntactically most prominent sentence constituent (e.g., Subject), and thereby trigger the corresponding syntactic choice. In one such study [15] participants viewed and described an animation film depicting one fish eating another fish. Participants’ attention was directed to one of the fish by means of an explicit (i.e., consciously processed) visual pointer presented above it. When participants’ attention was directed to the agent fish, active voice sentences (e.g. *the blue fish ate the red fish*) were by far the most likely types of descriptions; when attention was directed to the patient fish, passive voice descriptions (e.g. *the red fish was eaten by the blue fish)* were predominantly articulated. The authors concluded that the (explicitly) visually cued referents were more likely to be assigned the subject position in an English transitive sentence. In another study by [7] using implicit visual cues, participants were more likely to produce sentences in which the cued referent appeared in the sentence-initial slot, acting typically as its Subject. Finally in a study by [13], speakers’ syntactic choice was analysed under combinations of syntactic, lexical, and visual priming manipulations. Adding to the previous studies, visual cueing effects were registered both in the presence and in the absence of concurrent linguistic manipulations (syntactic priming and lexical priming) suggesting that syntactic choice may be an integral product of information biases derived simultaneously from different information inputs. This accumulating psycholinguistic evidence is further corroborated by neural simulations and neuroimaging studies showing an intimate link between attention and naming [17–20].

#### Multimodal and cross-modal attention

The evidence reviewed above provides important support to the idea that referent-directed visual attention plays a distinct role in the syntactic selection process. At the same time, psycholinguistic research on the role of attention in sentence production in general, and in motivating syntactic choice in particular, has been limited to manipulations of visual spatial attention and, correspondingly, to the use of the visual cueing paradigm. While the visual modality undoubtedly dominates during event perception, adequate understanding of a perceived event has to rely upon input from other perceptual modalities as well (e.g., auditory and motor). As a result, attentional contribution to the sentence production process may result from a selective process that filters information from other perceptual modalities in addition to the visual one. From this point of view, an investigation of whether the attentional system collects available sensory input from individual modalities and integrates them as a general attentional bias toward one of the available syntactic frames is necessary.

This “generalist” view of attention is supported by a number of studies on cross-modal attention showing that spatial attention can be successfully manipulated by e.g. tactile [21–26] and auditory cues [27–30]. For example, a recent study by [30] showed that a lateral *auditory* cue modulated the emergence of a lateral affordance effect that has been previously repeatedly shown to emerge from *visual* perception of manipulable objects. Instead of using visual cues, [30] cued their participants towards a manipulable object’s (e.g. kitchen appliances) handle with an auditory cue. Participants gave faster responses when the location of the cue and the direction of the handle were congruent with the response button. Similarly, a study by [29] showed that unimodal (visual and auditory), as well as multimodal (audiovisual) attentional cues are both capable in capturing visuo-spatial attention. Furthermore, similar results have been obtained for a combination of auditory, tactile and audio-tactile cues [31]. These and other studies clearly show that auditory and motor cues as well as visual cues can successfully orient spatial attention.

In addition, neuroimaging studies clearly show that the human brain simultaneously processes inputs from multiple input modalities. Attention in this case plays the role of a filter, deciding which sensory input should be prioritised [32]. This system integrates both the top-down control processes, such as the current goals, but also bottom-up processes, such as the saliency of the perceived stimuli (e.g., [33]), for a description of a saliency map and its role in shifts of visual attention). Importantly, the strength of the resulting attentional bias may differ depending on the specific modality of the sensory input. Animal studies confirm this *asymmetry* of the interplay between visual attention on the one hand and auditory and motor attention systems on the other. More specifically, they suggest that there is a more intimate link between visual and motor attention than between visual and auditory attention [34]. TMS and fMRI studies (see [35], for a review) present evidence that the organisation of the posterior parietal cortex of the human brain is quite similar to that of primates, thus suggesting that the relation between attentional modalities in humans may resemble that of a primate’s brain.

Indeed, a number of neuroimaging studies confirm the existence of a highly automatic multifaceted attentional system based on integrating input from different modalities (e.g., [36–38]). This is further supported by ample neuroimaging evidence confirming the existence of mechanisms for multimodal sensory integration in the human brain (e.g. [3, 39, 40]). Put together, the psycholinguistic and the neuroimaging evidence reviewed thus far points to the possibility that there can be a single cross-modal attentional mechanism underlying syntactic choice during sentence production. If so, then both motor and auditory attentional cues should influence syntactic choices in a similar fashion as has previously been documented for visual attentional cues. This suggestion, however, has not been experimentally tested so far.

To investigate the link between motor and auditory attention on the one hand and syntactic choice on the other, the present study examined whether auditory and motor lateral cues can affect syntactic choice similarly to the previously reported visual cue effects. English speaking participants described extemporaneously visually presented transitive events involving two referents, an agent and a patient. Their attention was directed towards the location of one of the interacting referents by means of either an *auditory* or a *motor* cue. As briefly reviewed above, a universalist view of attention and sensory integration suggests that lateral attention can be directed by means of auditory and motor as well as the visual cues. As such, our study focuses on whether the grammatical choice can, in principle, be biased by auditory and/or motor attentional cues as well as by the visual ones. This general goal motivated our choice of specific cueing manipulations (see in detail below). Other studies (e.g. [10]) used attentional cueing manipulations that, arguably, provide a more direct approximation to real-life discourse situations, such as interlocutors’ gaze cues, which was not a goal of this paper. We analysed the proportion of passive-voice responses as a function of cue location and cue modality. Of particular interest to us was whether attentional cues originating from the motor and auditory modalities can bias syntactic choices in a manner comparable to visual attentional cues used in earlier studies.

## Method

The study has been approved by Northumbria University ethics committee.

### Design

Two factors were orthogonally manipulated: Cue Type (the cue was provided in the *auditory* or in the *motor* modality) and Cue Location (attention was directed towards the *agent* or towards the *patient* of the depicted event for description). This resulted in a 2x2 factorial design with Cue Type and Cue Location as within-subjects/within-items factors. The dependent variable was the proportion of passive voice event descriptions.

### Participants

24 participants (16 females, mean age = 21.7, SD = 2.87) were recruited from the population of students and staff at Northumbria University. To participate in the study, participants had to be monolingual native English speakers, have normal (or corrected to normal) vision, and have no language or attention-related disorders (e.g., dyslexia, ADHD). Participants received course credits or £5 subject remuneration for their participation. All participants gave written informed consent before taking part.

### Materials

**Fig 1:**
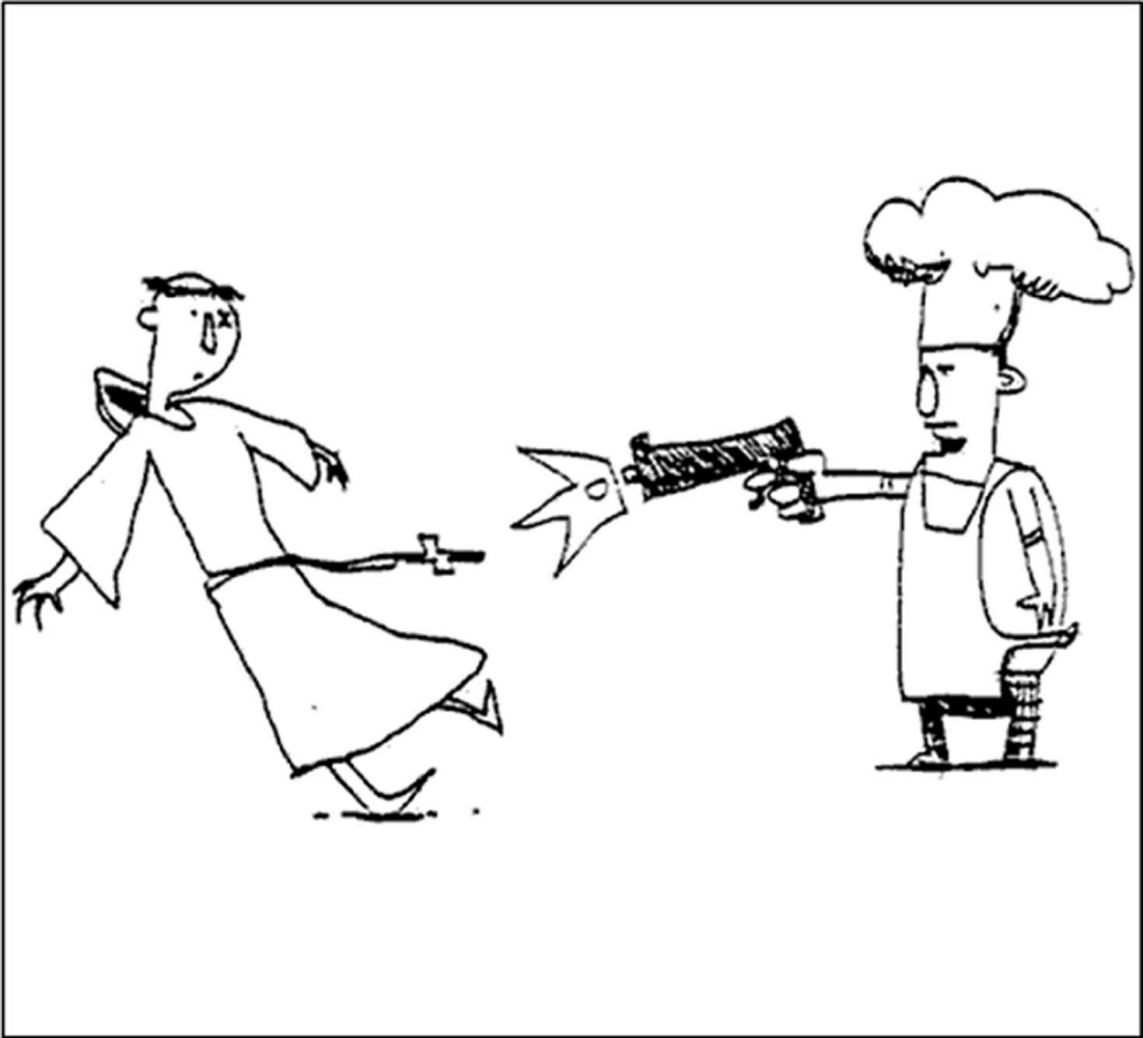
Stimulus example. A transitive event “The monk is shot by the chef”.

Picture materials including character names, event names, stimuli and filler pictures were adopted from the study by [13] Target pictures depicted six transitive types of events (*hit*, *shoot*, *chase*, *touch*, *push*, *kick*) rotated across sixteen referents (*artist*, *chef*, *clown*, *cowboy*, *monk*, *nun*, *pirate*, *policeman*, *swimmer*, *dancer*, *professor*, *waitress*, *burglar*, *boxer*, *and soldier*). Eight pairs of different characters interacted in each of the six event types, giving 48 transitive-event pictures in total (for an example see Fig 1). There were equal numbers of agent-on-the-left and agent-on-the-right event orientations. Also included were 98 filler pictures, depicting non-transitive events. Materials were presented in a pseudo-random order such that a minimum of two filler pictures separated the target pictures from each other. Materials were arranged into four lists, which allowed all events to feature in all four experimental conditions.

### Apparatus

The experiment was implemented using SR Research Experiment Builder v1.6.121 programming environment (SR Research Ltd, Ottawa, Canada). An EyeLink 1000 Desktop eye-tracker (SR Research) was used to control the participant’s gaze allocation. A Clear view 17-inch display with a refresh rate of 60Hz was used. Eye movements were recorded from the right eye only with a 1000 Hz sampling rate. Auditory cues were delivered by Genius E120 Desktop Speakers via ASIO sound card. The speakers were positioned to the left and to the right of the monitor 15cm from the centre of the screen to roughly approximate the source of the cue to the location of the cued referent (cf. [26, 29, 31]). At the same time, the spatial correspondence between the cue and the event’s referents was not a priority in this study in that we did not attempt to set-up an experimental setting ecologically proximal to a real-life discourse situation similar to a gaze cue, for example, used in other studies (cf. [10]). Generated sentences were recorded using a voice recorder application (Voice Record PRO v.3.2.2, VENDOR) and stored on a password protected PC. Participants were seated 60 cm away from the monitor with their head position controlled by a chinrest. Participants were instructed not to force their head on the chinrest, so they could speak normally.

### Procedure

The study took place in the eye-tracking laboratory on Northumbria University campus. On arrival, participants provided their demographics and signed consent forms. After reading experimental instructions, participants first went through a practice session and calibration (5–10 minutes depending on the ease of calibration). During the practice session, participants were engaged in two tasks. First, they familiarised themselves with the 16 referents that were to appear in the main experiment: The character’s depictions were sequentially presented centrally on screen, with their names written underneath. Participants simply read the character names aloud. This ensured that participants familiarised themselves with the referents’ appearances and names, and minimised the chances that they would find the referents difficult to recognise and describe. Also, this procedure reduced potential ambiguity in naming referents (e.g., *monk*, *priest*, *clergyman–*for the character “monk”). Second, participants practiced describing events similar to the ones they would later encounter in the main experimental session. Participants saw fourteen randomly selected events in a randomised order, with each picture depicting an event with one or two referents (previously practiced) and its name (e.g., *chase*) written underneath. As before, participants were instructed to examine the event and read its name aloud. The purpose of the event practice session was to minimise the variability of potential lexical candidates for the event description (e.g., *punch*, *beat* for the “hit” event), especially since previous research has shown that different verbs have different passive-vs-active voice distributions in corpora [33] The participants were also instructed to keep descriptive detail in their responses to a minimum (i.e. to produce simple descriptions like “The pirate kicks the boxer” rather than “The pirate with one leg forcefully kicks the boxer, and the boxer will probably fall over because of that”).

Upon completion of the practice session, participants received instructions for the main part of the experiment. Participants were instructed that they would be presented with either an auditory cue (AC, auditory signal) or a motor cue (MC, coloured circle), respectively.

In AC trials, an auditory signal (a tone of 500 ms duration, sample rate 22050 Hz) was presented laterally either from the left speaker or the right speaker. Participants were instructed to direct their gaze to the central fixation point (black dot in the middle of the screen) and wait. As soon as a stable fixation was confirmed by the fixation trigger (gaze contingent offset after 150 ms fixation maintenance), the lateral auditory cue was played. Participants were instructed to expect to hear the cue from either left speaker or right speaker; the distance between the speakers was sufficient for participants to be able to discriminate the origin of its location.

**Fig 2:**
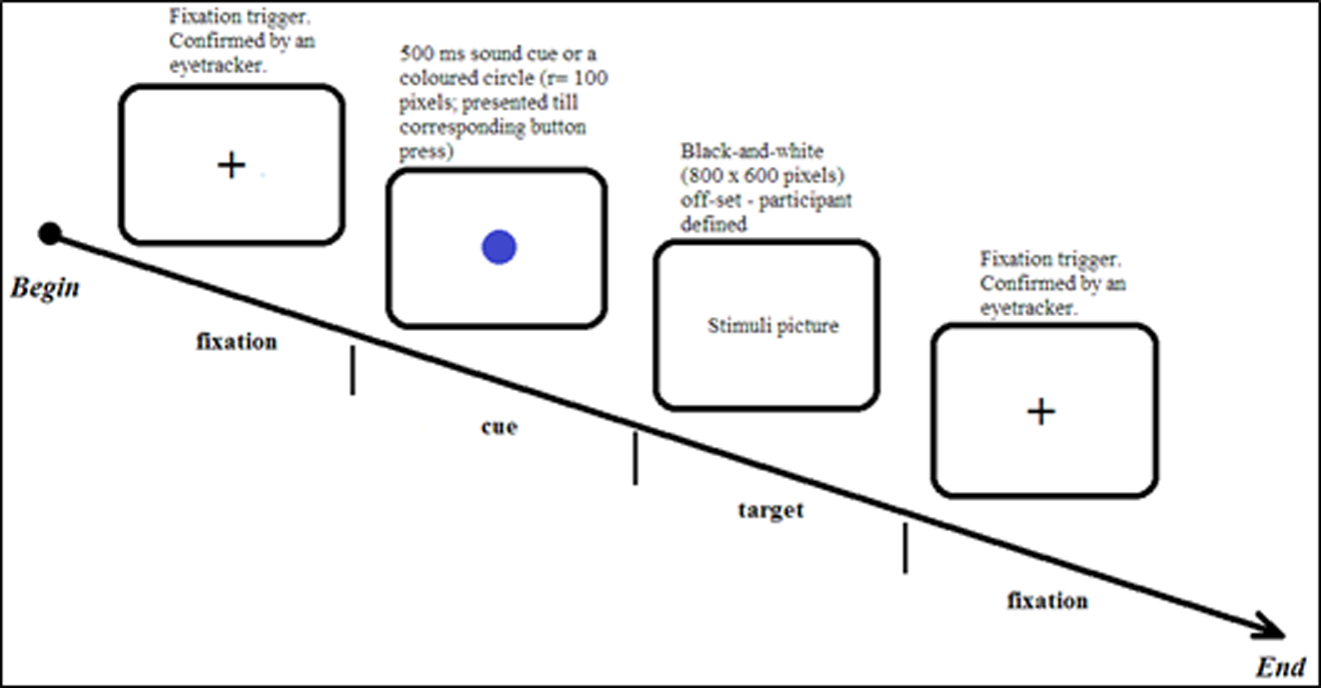
A diagram of the experimental procedure.

In MC trials, participants were instructed to press the green or the blue key depending on the colour of a central circle that was presented after the central fixation dot. These two colours were assigned, in a counterbalanced fashion, to two keyboard keys. The ‘Z’ key was used in the leftward cue condition (to be pressed with the index finger of the left hand) and the ‘NumPad2’ key for the rightward cue condition (to be pressed with the index finger of the right hand). Participants were instructed to rest their index fingers on these keys to avoid visual search for the key locations. For half of the participants, if the colour of the central circle was green (colour code #22B14C), participants had to press the left (‘Z’) key to progress, if it was blue (colour code #3F48CC), they had to press the right (‘NumPad2’) key. For the other half, this mapping rule was reversed. This was followed by the target event presentation (see Fig 2 for a visualisation of a trial).

The main task was to describe the event in one sentence mentioning both characters and their interaction. On completion of each trial, participants proceeded to the next trial by pressing Space.

Finally, participants were presented with a short training session (8 trials). During this session, participants familiarized themselves with the procedure and the experimental task including differentiating the location of an auditory cue (left vs. right) according to the specific color-key mapping rule, which participants were able to do correctly; finally, practice session allowed participants to practice their responses.

### Data analysis

The audio recordings of the event descriptions were transcribed and coded as either Active Voice or Passive Voice. To be coded as Active Voice, the description had to include a subject noun phrase referring to the agent, followed by a transitive verb describing the event, and an object noun phrase referring to the patient (e.g. *The pirate is chasing the boxer)*. To be coded as Passive Voice, the description had to include a subject noun phrase referring to the patient, followed by a passivised transitive verb describing the event, and a by-phrase referring to the agent (e.g. *The boxer is being chased by the pirate)*. Conjoined noun phrases (e.g. *The boxer and the pirate are running)*, truncated passives (e.g. *The boxer is being chased*), incomplete or ungrammatical sentences, and multi-sentence responses were excluded from the analysis, affecting less than 1% of the data.

Inferential analyses were performed using Generalised Linear Mixed Effects Models (GLMM), as part of the lme4 package in R. The dependent variable of interest was the occurrence of a Passive Voice description (True = 1, False = 0), and therefore a *binary logistic* model was specified in the family argument of the glmer() function. The model included a full-factorial Cue Modality (Auditory, Motor) × Cue Location (Agent, Patient) fixed effects design. All predictors were mean-centred using deviation-coding. Following [41] the maximal random effects structure justified by the design was used. Since the two experimental variables were both within-subjects and within-items, the model therefore included by-subject and by-item random intercepts as well as by-subject and by-item random slopes for every main effect and interaction term in the fixed effects design. Random correlations were also included. P-values were obtained via Likelihood Ratio Chi-Square (_*LR*_*χ2*) model comparisons.

## Results

**Fig 3:**
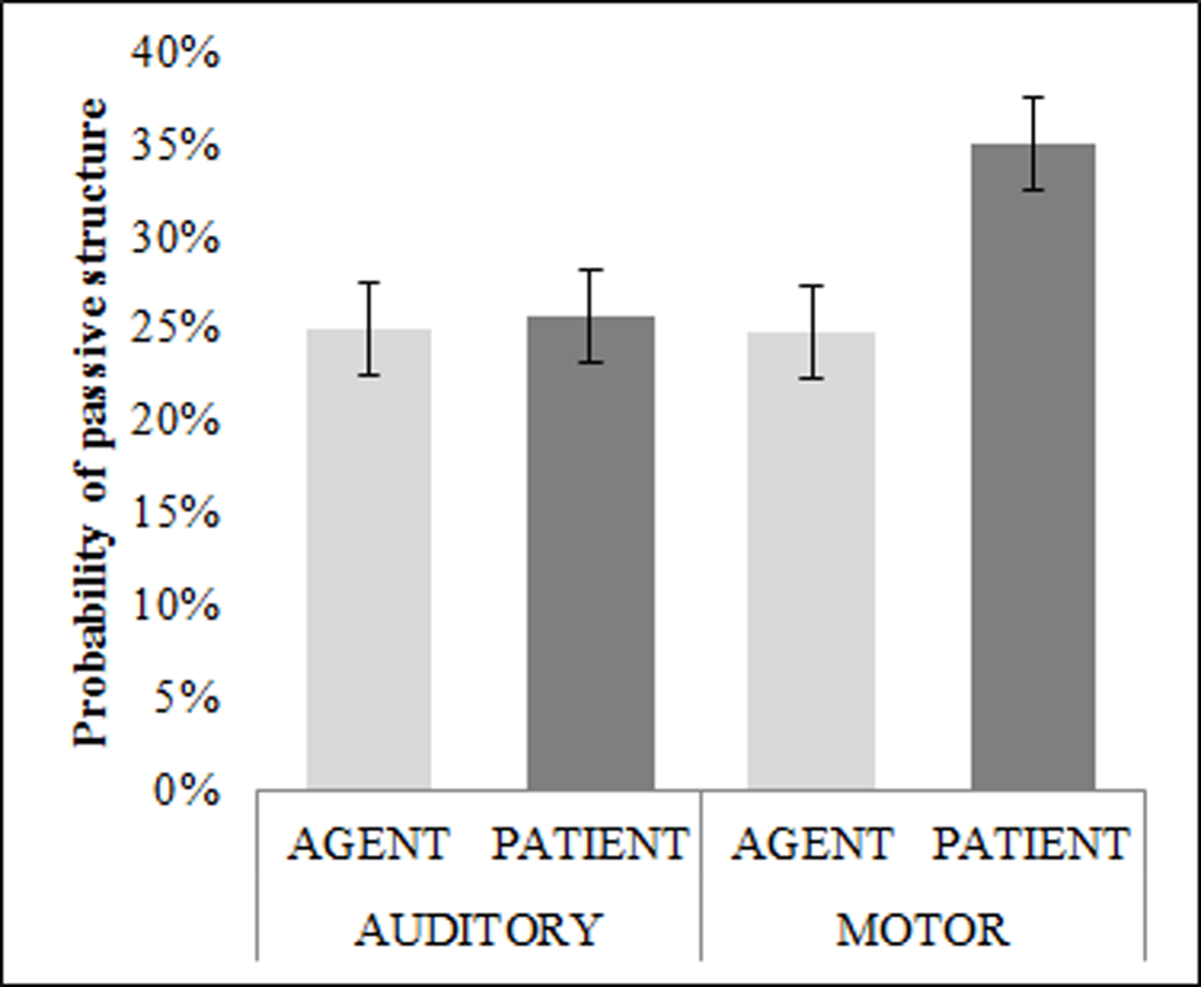
Group-average probability of passive-voice selection as a function of Cue Location. Error bars represent standard errors of means.

Table 1 shows the distribution of active versus passive voice responses across experimental conditions. As can be seen from the table, particularly in the motor modality, patient-cueing resulted in increased proportions of passive voice responses at the expense of active voice responses. The grand average intercept of the GLMM was estimated as −1.325 log odds units (*SE* = 0.251), which is considerably below zero (i.e., smaller than 0.5 in probability space). This is because passive-voice responses were greatly outnumbered by active-voice responses overall, a result that is in line with previous experimental findings (see [1], for a review) as well as with corpus-data (e.g. [42]). In terms of experimental influences, there was a reliable main effect of Cue Location (_*LR*_*χ2*(1) = 5.299, *p* = .021) due to more passive-voice descriptions in the Patient-Cued than in the Agent-Cued condition. Crucially, while the main effect of Modality was not significant (_*LR*_*χ2*(1) = 0.641, *p* = .423), there was a reliable two-way interaction (_*LR*_*χ2*(1) = 4.940, *p* = .026), indicating that the effect of Cue Location was dependent on Cue Modality (see Fig 3). Simple effect analyses (based on treatment-coding of the Cue Modality predictor) confirmed that the effect of Cue Location was significant only in the Motor modality (*b* = .743, *SE* = .256; _*LR*_*χ2*(1) = 8.010; p < .005) but not in the Auditory modality (*b* = .021, *SE* = .193; _*LR*_*χ2*(1) = 0.012; p = .914). Therefore, it can be concluded that attentional cues in the motor modality, but not in the auditory modality affected participants’ syntactic choices for event description.

**Table 1:** Probabilities of active versus passive voice responses across all participants and trials.

Cue Modality	Cue Location	Active Voice	Passive Voice
Auditory	Agent	.750 (432)	.250 (144)
	Patient	.743 (428)	.257 (148)
Motor	Agent	.752 (433)	.248 (143)
	Patient	.649 (374)	.351 (202)

Absolute cell counts in brackets by levels of Cue Modality (Auditory, Motor) and Cue Location (Agent, Patient).

## Discussion

The aim of this study was to investigate whether motor and auditory lateral attentional cues affect syntactic choice during visually mediated sentence production. Previous research has shown that manipulation of attention by visual modality cues leads to the assignment of the cued referent to the Subject position in a spoken sentence, thus triggering the choice of syntactic structure. Furthermore, neuroimaging and cognitive studies suggest that attentional modalities have common control mechanisms, but due to differences in processing might have variations in the way they affect syntactic choice. Overall, our results show that *motor* cues affect syntactic choice in a manner similar to visual cues, indicating the existence of a partially multimodal attentional mechanism affecting syntactic choice in sentence production. However, the same was not true for the auditory cues. Below, we discuss the implications of this main finding.

First, the results of the present study provide further support to the existing research on the role of attention in biasing syntactic choice during sentence production. Our data show that participants were more likely to describe target events by using passive-voice sentences when the previously presented cue was in the patient location. This replicates the pattern of results from studies of visual attention and sentence production studies [11, 13, 15,16]. Thus, cueing attention before sentence production is an important factor affecting the grammatical structure of the sentence.

Secondly, our data reveal, for the first time, that this effect is not limited to the visual modality, since priming the speaker’s attention via lateral motor cues led to a similar priming effect, as confirmed by a simple-effect analysis of the reliable interaction between Cue Type and Cue Location. The same analysis revealed that the main effect of Cue Location was carried almost exclusively by motor-modality cues, while proportions of passive-voice descriptions in the auditory-cue trials were not reliably affected by Cue Location. This suggests constraints on the scope of any cross-modal components in the interface between attention and syntactic choice especially if we take into account the efficiency of auditory cueing in driving attention in general [26, 29–31]. One further possibility is to understand whether the same pattern of results can be achieved in less strict word-order languages (e.g. Russian, Finnish, etc.). There have been studies of visual cueing in Russian [43], Korean [8] and Finnish [12] which show somewhat conflicting results. Whereas in Russian, syntactic choice was biased by visual cueing, no syntactic structure alterations were observed in Korean and Finish, although the attentional manipulations were successful as such. Possibly, other attentional modalities can have a similar effect on syntactic choice in different languages.

Finally, the difference between the auditory and the motor cues’ capacity to affect syntactic choice in our study may result from the intrinsic differences between the auditory and motor cues we used. The capacity to laterally displace attention is generally different for these two modalities with auditory cues being less powerful in this respect [44]. This is partially explained by the fact that, in the auditory modality, the location of the cue source is extracted from the signal differences between the two cochleas in the tonotopically organised auditory cortex, making the successful cue processing easier [45] thus making it a non-binary cue with multiple possible location outcomes. Conversely, in order to process motor cues similar to the ones used in our study, one has to process the color of the cue and then make a decision regarding the required key press before discriminating it as a binary go/no-go cue. As a result, the auditory cue may be less successful in biasing syntactic choice simply because it requires less processing and thereby does not lead to the establishment of a strong lateral attentional bias. Of course, our results cannot be taken to mean that auditory attention does not affect syntactic choice whatsoever. What our results do indicate, however, is that motor modality cues are substantially more effective than auditory cues in attracting attention to visual referents and affecting syntactic choice (as reflected in the reliable interaction between Cue Location and Cue Modality). This opens a possibility to further study the relation between attentional modalities.

Neuroimaging studies suggest, that although some control mechanisms are shared between cues from different attentional modalities, orienting mechanisms differ. Arguably, it is possible to shut down or enhance orienting of attention; for example, repetitive TMS of right angular gyrus caused interruptions in orienting of attention [46], while a 1Hz synchronised TMS of left or right parietal cortex for 10 minutes significantly improved subjects' attention to ipsilateral targets [47]. Thus, the present hypothesis may be further tested by using TMS in languages where there is seemingly no effect of cueing on syntactic choice, such as Finnish or Korean. Stimulation or inhibition of the above brain regions can possibly cause speakers of such languages to switch syntactic structure towards the cued referent and thus it can help generalising this mechanism between languages with different levels of strictness of word order.

## Conclusions

To describe a picture in a particular way, an interplay between a number of linguistic and nonlinguistic factors needs to be established. One of those factors is how attention is allocated towards the referents of the described event. Our study has shown for the first time this process can be affected not only by manipulating visual attention. Motor attention (e.g. lateral key presses) can bias the choice of syntactic structure by shifting attention towards the referent. In contrast, an auditory cue (a lateralised sound) did not generate the same effect, which may be due to a number of factors, such as the depth or time of cue processing or a closer link between visual and motor networks in the brain. Future research is necessary to address the multimodal attention mechanisms in syntactic choice in more detail, as well as to test their generalisability to other languages and to establish their underlying neural mechanisms.
